# Equitable Open Access

**DOI:** 10.1038/s44319-025-00655-7

**Published:** 2025-12-08

**Authors:** Bernd Pulverer

**Affiliations:** European Molecular Biology Organization, Meyerhofstrasse, Heidelberg, 69117 Germany

**Keywords:** Science Policy & Publishing

## Abstract

The predominant financial model for OA is based on Article Processing Charges (APCs), which often forces authors to assemble support from diverse sources, including research grants. For a sustainable transition to equitable OA, funders could award funding to journals on a competitive basis to encourage adoption of open science, reproducibility and quality mandates.

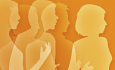

Around half of all scientific papers are now published Open Access. This represents a remarkable transition from a subscription-based publishing model set in motion with the ‘Berlin declaration’ nearly two dozen years ago. However, the transition has slowed and an increased urgency is evident in discussions on how to convert ‘the other half’ (e.g., https://oa2020.org/b17-conference/; https://royalsociety.org/science-events-and-lectures/2025/07/future-of-scientific-publishing/).

A priori, having half the literature fully accessible would appear to be a laudable achievement, but the devil is in the details. First, OA is not symmetrically distributed across geography, institutions, funders and—for want of a better word—quality. We are still a considerable way off sharing the majority of the most significant findings OA. Second, not all OA is equal: many funders have decided to effectively forego the original goal—the open publication of the peer-reviewed, typeset ‘version of record’ papers (‘Gold OA’)—to allow so-called ‘Green-OA’, where a pre-final version of the work is archived. In the form of preprints, Green OA is a powerful accelerator of research progress and efficacy, yet the intention of preprints is certainly not to replace the quality controlled, editorially enriched ‘version of record’ (Pulverer and Lemberger, 2019). Parking pre-final versions of research papers in a fragmented landscape of local repositories, some with undefined sustainability and public access, can be a costly diversion from the goal of a universally accessible, quality-controlled scientific record.

The third issue is that the transition period to full OA is inherently costly: so-called ‘hybrid journals’, which host both OA and subscription content, were an excellent interim solution but, as a permanent fixture, they risk partially double charging the community for subscription and OA, as both are not neatly separable due to finnicky multi-year publishing agreements.

Contrary to some speculation, the Article Processing Charge (APC) model was not invented by for-profit publishers to amplify profit margins; rather, it was encouraged by the OA community as a transparent open-market mechanism, where authors would intentionally choose a reasonable value proposition. This, of course, assumes ‘standard’ market forces, where customers make choices based on objective criteria of quality and value, which is manifestly not the case while journal choice is dominated by research assessment considerations.

A one-size-fits-all APC model cannot be equitable in a diverse global research landscape, but the argument that APCs are *ipso facto* inequitable is wrong. There is no reason in fact why authors have to be tasked with managing publication cost—just as they did not manage subscription agreements. Institutional services should support scientists and OA publishing agreements can ensure that the market power that comes with a large customer base buffers profit maximization of commercial publishers. This consolidation started to happen with widespread ‘transitional agreements’, but these derived from subscription budgets and—counter intuitively—typically *exclude* the desired outcome of the ‘transition’: fully OA journals (like this journal). This is as short sighted as it is damaging by giving OA via hybrid journals a clear market advantage, thereby discouraging full OA conversion. Germany and the UK, among others, demonstrate that more inclusive ‘deals’ can be achieved with Projekt DEAL and JISC, respectively. Equity is also possible at the level of individual APCs, as charges can be adapted to reflect research investment across nations and subjects. For example, EMBO Press is part of a 5-banded APC scheme based on GDP and research funding. If this is matched with a robust discount and waivers policy (at EMBO Press, we will not exclude any author due to an inability to pay), APCs can provide a fair mechanism.

Only two other approaches to OA show some promise: journals moving from subscription to full OA may be able to convince existing subscribers to continue as before: this ‘subscribe-to-open’ model is certainly community spirited and has been shown to work in principle. In contrast, ‘Diamond OA’ relies on direct subsidy of journal costs to provide free and full access to readers and authors alike. The money could come from philanthropy, a public funder, a research institution or the owners of a journal. Such an asymmetrical investment can certainly be justified—at least for those journals which provide a key service to the scientific enterprise. A caveat is that questions may arise about market distortion if public finances are used. A key limitation of Diamond OA is the lack of scalability across the quality literature and, while diamonds are forever, most philanthropy is not. Consider also that single-source income can come with influence on editorial processes. Initiatives like the European Diamond capacity OA hub are attempting to covert Diamond OA into a scalable, sustainable model, but have yet to deliver globally relevant solutions.

Is there a scalable alternative to APCs, repurposed subscription budgets or Diamond OA which would support the scientific process optimally? Indeed, there is: competitive funding for journals. Competitive grants, judged by subject experts, are at the heart of the scientific process, so why not extend this tried and tested principle to journals? Such grants would set clear and measurable evaluation criteria based on specific services deemed to enhance the scientific process, such as transparent peer review, quality control and open-science processes. They would be open to application by any journal and require evidence for such quality attributes.

In our view, such a mechanism would not in fact need to fund the entire journal revenue. Rather, it could be restricted to costs associated with auditable policies that a funder already encourages or mandates. Thus, funding criteria could include policies to enhance reproducibility and open sharing, underpinned by quality control, research integrity and open-science services mandated by a journal. It might also include funding for journal content that is valuable for the scientific community or of public interest, but not directly supported by APC-based mechanisms, such as Reviews, Commentaries and journalistic articles (at this journal, this includes the entire ‘Science & Society’ section).

The baseline cost of a journal, that is editorial selection, peer review and production, would continue to be covered by APC charges independently of such a grant. This would align the costs of those journals that go above and beyond the *status quo* with journals that don’t feature such quality attributes. Such a ‘hybrid Diamond OA’ mechanism would thus be more scalable, as funders would not carry the full costs, while encouraging journals to adopt the strategic policies of the grant provider. At the same time, the baseline APC would remain affordable for all authors.

Scientific journals not only serve to enhance and filter research, they are also gatekeepers for science policy. Funders increasingly set policies to enhance quality and open sharing, yet struggle to police compliance. By encouraging a consistent set of policies ‘from both ends’, funders would benefit by encouraging compliance. Authors benefit by working to a consistent set of policies. It would also benefit OA journals with a high level of quality control and selectivity, which typically translates into higher publication charges.

In this issue, Tautz and Rainey (2025) go beyond grants to argue for standardized evaluation procedures for scientific journals, noting that any public payments for Open Access fees ’should be conditional upon journal accreditation against transparent, enforceable standards’. To implement such a mechanism, Tautz and Rainey propose ‘clearinghouses for journal evaluation and certification, that establish accreditation and reputation criteria based on transparent, standardized benchmarks’, rather than relying on funder based expert review. They argue this ‘would generate a mechanism for grant systems that would finance journals according to diamond open-access standards as a basic infrastructure for science’.

Sustainable OA at high quality remains a challenge for selective journals, but cutting corners to lower costs would be a dramatic failure, as it would directly undermine their value to the community. Is it reasonable to expect the relatively low fraction of accepted authors to support the costs of rejecting lower-quality research or publishing other article types they may not always be particularly interested in? Competitive grants for Gold OA journals and formal evaluation of journal quality would both present scalable solutions to support those journals which can demonstrably add value to the scientific community.

The scientific process will only work effectively if funders (upstream) and Journals (downstream) set consistent policies. What better way to catalyze this synchronization than by awarding funding and by setting transparent criteria for a more reliable, fair and open research output informed by the scientific community for a new accreditation institution? The EMBO scientific publications certainly are ready to apply to any such initiative.

## References

[CR1] Pulverer B, Lemberger T (2019) Peer review beyond journals. EMBO J 38(23 Dec):e103998. 10.15252/embj.2019103998 31788827 10.15252/embj.2019103998 PMC6885733

[CR2] Tautz D, Rainey PB (2025) An evaluation system for scientific journals. EMBO Rep. 10.1038/s44319-025-00649-5 10.1038/s44319-025-00649-5 PMC12795802 41361691

